# Antibodies against gonadotropin-releasing hormone (GnRH) in patients with diabetes mellitus is associated with lower body weight and autonomic neuropathy

**DOI:** 10.1186/1756-0500-6-329

**Published:** 2013-08-17

**Authors:** Kerstin Berntorp, Anders Frid, Ragnar Alm, Gunilla Nordin Fredrikson, Klas Sjöberg, Bodil Ohlsson

**Affiliations:** 1 Department of Clinical Sciences, Division of Endocrinology, Skåne University Hospital, Malmö, Lund University, Lund, Sweden; 2 Department of Clinical Sciences, Experimental Cardiovascular Research Unit, Skåne University Hospital, Malmö, Lund University, Lund, Sweden; 3 Faculty of Health and Society, Malmö University, Malmö, Sweden; 4 Department of Clinical Sciences, Division of Gastroenterology, Skåne University Hospital, Malmö, Lund University, Lund, Sweden; 5 Department of Clinical Sciences, Division of Internal Medicine, Skåne University Hospital, Malmö, Lund University, Lund, Sweden

**Keywords:** Autoantibodies, Diabetes mellitus, Esophageal dysmotility, Gastroparesis, Gonadotropin-releasing hormone (GnRH), Secondary complications

## Abstract

**Background:**

Esophageal dysmotility and gastroparesis are common secondary complications in patients with diabetes mellitus. Patients with dysmotility express antibodies against gonadotropin-releasing hormone (GnRH) in serum. The aim of the present study was to scrutinize patients with diabetes mellitus with regard to the presence of GnRH antibodies, and to examine associations between antibodies and clinical findings.

**Results:**

Thirty-nine consecutive patients with diabetes mellitus were included in the study after clinical examination and examination by esophageal manometry and gastric emptying scintigraphy. Serum was analyzed for the presence of antibodies against GnRH using an ELISA, and values are expressed as relative units (RU). Two age- and gender-matched healthy subjects per each patient served as controls. The prevalence of IgM GnRH antibodies in patients was 33% compared to 14% in controls (p = 0.027), with a higher antibody titer; 1.2 (0.6-5.0) and 0.2 (0.1-0.3) RU, respectively (p = 0.000). The expression of IgG antibodies was 15% in patients and none in controls (p = 0.000). Lower body mass index was associated with the presence of IgM antibodies (OR = 0.835, 95% CI = 0.699–0.998), and autonomic neuropathy with the presence IgG antibodies (OR = 9.000, 95% CI = 1.327–61.025). Esophageal dysmotility (69%) or gastroparesis (18%) were not associated with the presence of IgM antibodies (OR = 0.589, 95% CI = 0.143–2.424 and OR = 3.407, 95% CI = 0.633–18.350, respectively). Neither was esophageal dysmotility associated with IgG antibodies (OR = 2.500, 95% CI = 0.259–24.096).

**Conclusions:**

Antibodies against GnRH are more common in patients with diabetes mellitus compared with healthy controls. IgM antibodies are associated with lower body mass index and IgG antibodies are associated with autonomic neuropathy.

## Background

Gastrointestinal dysmotility is a common disorder in the population, especially as a complication secondary to diabetes mellitus [1-3]. Recently, we have shown that 63% of patients with diabetes mellitus suffer from esophageal dysmotility and 13% from gastroparesis [4]. Although some patients suffer from gastrointestinal symptoms, such as regurgitation, vomiting, and abdominal fullness and bloating after eating, the associations between symptoms and objective findings are poor [2-4]. The etiology of gastrointestinal dysmotility needs to be elucidated. Damage to interstitial cells of Cajal, loss of neuronal nitric oxide expression, hyperglycemia, vagal or autonomic neuropathy, myopathy, and abnormalities in plasma levels of several hormones have been proposed [1,5].

Gonadotropin-releasing hormone (GnRH) is known to bind to specific receptors on the pituitary, controlling the secretion of the gonadotropins [6,7]. However, there is growing evidence for its role as a neurotransmitter in the enteric nervous system (ENS) [8], where it has been shown to inhibit gastric secretion and gastrin release [9], stimulate enteric motility [10,11], and inhibit cell proliferation in gastric epithelial and smooth muscle cells [12,13]. We have recently shown that treatment with GnRH in man and rats may lead to loss of enteric neurons, with ensuing development of gastroparesis and severe dysmotility [14,15]. Antibodies against GnRH have been observed in patients with irritable bowel syndrome (IBS), motility disorders, diabetes mellitus, and patients with functional bowel symptoms secondary to primary Sjögren’s syndrome. Patients with organic gastrointestinal diseases such as inflammatory bowel disease, celiac disease, and scleroderma did not express GnRH antibodies [16,17]. Thus, patients with diabetes mellitus frequently express antibodies against GnRH and secondary esophageal dysmotility, but the association between these two findings has to date not been examined [4,17]. The aim of the present study was therefore to further examine the prevalence of antibodies against GnRH in patients with diabetes mellitus, with or without esophageal dysmotility or gastroparesis, to further characterize the role of the antibodies and to search for possible associations with clinical findings.

## Methods

This study was performed according to the Helsinki Declaration and was approved by the Regional Ethics Review Board of Lund University. All subjects gave their written, informed consent before entering the study.

### Patients

The inclusion criterion of the study was diabetes mellitus in patients above 18 years of age. The first patient at each consultation occasion who fulfilled this criterion when scheduled for routine clinical follow-up at the Department of Endocrinology, Skåne University Hospital, Malmö, from January 2008–February 2010, was invited to participate in the study. Exclusion criteria were severe renal failure demanding dialysis or severe cardiac diseases. At the time of the inclusion, they completed an established questionnaire with 15 symptoms related to disturbances of the gastrointestinal tract (loss of appetite, swallowing disturbances, meal-related cough, early satiety, nausea, vomiting, weight loss, abdominal fullness, bloating, regurgitation, constipation, diarrhea, evacuation incontinence, symptomatic postprandial hypoglycemia and postprandial perspiration), previously used for these patients [2-4]. Types and duration of diabetes mellitus, presence of diabetes complications such as retinopathy (based on fundus photography), angiopathy, microalbuminuria (measured as the albumin/creatinin quotient), albuminuria, peripheral neuropathy (examined by patellar and achilles tendon reflexes, sensitivity to vibration and monofilament test), autonomic neuropathy according to established clinical criteria (sexual dysfunction, profound sweating and orthostatic blood pressure), drug treatments, concomitant diseases, and body mass index (BMI) were all recorded by the physicians.

Glycosylated hemoglobin (HbA1c) was analyzed at the Department of Chemistry, according to clinical routines. HbA1c values were collected as Mono-S and subsequently converted to the National Glycohemoglobin Standardization Program (NGSP) standard by use of the following algorithm: 0.923 x HbA1c (Mono-S) × 1.345 = HbA1c (NGSP) [18]. Percentage HbA1c values were converted to the International Federation of Clinical Chemistry (IFCC) standard in mmol/mol according to the following equation: IFCC (mmol/mol) = ([NGSP (%)] - 2,152)/0.09148 [18].

The patients were referred to investigations by esophageal manometry and gastric emptying scintigraphy. If both examinations could not be performed, the patient was excluded from the study. Patients were also referred to esophagogastroduodenoscopy when clinically indicated.

Thirty-nine patients of the 55 invited completed the study and provided blood samples for analysis of GnRH antibodies, and were thus finally included in the evaluation. The most common reason not to complete the study was an inability to swallow the manometry catheter.

### Esophageal manometry

Standardized esophageal manometry was performed with an intraluminal, solid-state transducer system (Gaeltec Ltd, Isle of Skye, Scotland). Polygraph ID converter digitized the analog signal. The software was PolyGram NET (Medtronic-SynMed Medical, Stockholm, Sweden). All pressure values were expressed in mmHg and referred to atmospheric pressure. The manometry catheter was introduced through the nose and fluoroscopically positioned in the distal esophagus with the patient sitting in an upright position, which is the standard method at our laboratory. With the catheter in place, all participants were instructed to swallow 10 ml of a barium contrast medium (60% w/v). At least five barium swallows were recorded. The video fluoroscopic image and the manometry registration were mixed using a video output card (Medtronic-SynMed Medical) [2,3]. Patients who fulfilled one or more of the following five criteria in the esophageal manometry with abnormal results were considered to suffer from esophageal dysmotility: 1: Absence of peristaltic contraction in the esophagus (aperistalsis > 0%), 2: Mean peristaltic contraction amplitude < 30 or > 200 mmHg in the esophagus, 3: Percentage of simultaneous, non-propulsive peristaltic waves in the esophagus > 10%, 4: Speed of the peristaltic wave < 3 or > 6 cm/s in the distal esophagus, and 5: Resting pressure in the lower esophageal sphincter (LES) < 10 or > 30 mmHg. Normal peristaltic activity was defined as propulsive contraction waves with peak amplitudes between 30–200 mmHg and a speed between 3–6 cm/s [2,3,19,20].

### Gastric emptying scintigraphy

A test meal was prepared by adding tin colloid labeled with 30–50 Mbq of technetium-99 to an egg, which was whipped in a glass cup in a hot water bath until coagulated. The egg and a slice of toasted white bread were cut into pieces smaller than 1 cm × 1 cm and served with 100 ml water at 37°C. The meal was eaten within 5 min. Immediately thereafter, a large-field, double-headed gamma camera (Philips Skylight, Philips Medical Systems, Best, The Netherlands) was placed anteriorly and posteriorly parallel to the upper abdominal wall. The radioactivity was measured continuously (1-min frames) for 70 min starting immediately after meal ingestion. A Region of Interest (ROI) representing the stomach was created and the activity of the first frame was taken as 100%. The gradual decreasing radioactivity, measured as the number of radioactivity decays per minute (counts/min), was plotted against time. The time elapsed to reach a 50% decrease of the activity in the ROI (T_50_) was identified as the point at which this plot crossed the 50% value. The values of the radioactivity measured were corrected for the half-life of ^99m^Tc, and for attenuation by using the geometrical mean values of the decay curves obtained from the two gamma camera heads used. T_50_ > 2 standard deviations (sd) for healthy control subjects (70 min) was considered abnormal [21].

### Measurement of human antibodies against gonadotropin-releasing hormone

Blood samples were drawn from patients and the serum was separated and kept frozen at -20°C until analyzed. Analysis of anti-GnRH antibodies was carried out by an ELISA slightly modified on the basis of the results described in previous studies [8,16]. The wells of micro titer plates were coated with human GnRH (L7134, Sigma, St Louis, MO, USA) for an overnight incubation at 4°C and thereafter the plastic wells were blocked with 0.5% fish gel solution (G7765, Sigma) in PBS containing 0.05% Tween-20 (PBS-T). Serial dilutions of patient serum (1/100, 1/500 and 1/2500 in PBS-T) were then added to the plates and incubated for 2 h at room temperature (RT) and overnight at 4°C. After rinsing with PBS-T, deposition of autoantibodies directed to GnRH was detected using biotinylated rabbit anti-human IgM (673211, MP Biomedicals, Solon, OH, USA) or IgG antibodies (ab7159, ABcam, Cambridge, MA, USA) appropriately diluted in PBS-T. After another incubation for 2 h at RT, the plates were washed and the bound, biotinylated antibodies detected by alkaline phosphatase-conjugated streptavidin (405211, Biolegend, San Diego, CA, USA), incubated for 1 h at RT. To develop a color reaction a phosphatase substrate kit (37620, Pierce, Rockford, Ill, USA) was used. The absorbance at 405 nm was measured after 2 h of incubation at RT. A plasma pool from healthy blood donors was included on each ELISA plate for measurements of the variation. The plasma pool was used for the calculation of the intra-assay and inter-assay coefficient of variation, which was 11.5% and 16.1%, respectively, for IgM and 11.5% and 25.4%, respectively, for IgG. Antibody levels are presented as relative units (RU) (absorbance values after subtraction of background levels and multiplied by 100). Relative units over 0 were considered as a positive antibody level [17].

The controls were chosen from a cohort of healthy blood donors previously described in detail [17]. Over a period of five months (October 1996–February 1997), blood donors were offered antibody screening for gastrointestinal diseases. To be able to include all blood donors in Malmo, sera from male donors were collected over a 3-month period and from female donors over a 4-month period (in accordance with their regular donation intervals). A total of 1,970 donors were included. During this period, 2,135 blood donations took place, which means that at least 92% of donors agreed to be included. From this sample cohort, 50 men and 50 women from each 10-year age span period, between 20 and 70 years of age, were randomly included. As few blood donors above 60 years of age, only 16 women and 40 men were included in the age group 60–70 years. In total, 456 controls were examined during the same time period as the patients. From this cohort, two age- and gender-matched controls were randomly extracted for each patient in this study.

### Statistical analyses

The data were analyzed using the statistical software package SPSS for Windows^©^ (Release 20.0; IBM). All variables were analyzed for normal distribution by Kolmogorov-Smirnov test. Group-wise differences between patients and controls were tested by using the unpaired Student’s *t*-test and, when normality was rejected (antibody levels), the Mann-Whitney *U*-test was used. Fisher’s exact test was used for dichotomized variables. Correlations were calculated by Spearman rank correlation test. Values are expressed as mean ± standard deviation (SD) or median, interquartile range (IQR). Binary logistic regression analysis was performed to test for an association between the presence of antibodies against GnRH (dependent variable) and clinical findings in patients. Independent variables were age (years), gender, type of diabetes, duration of disease (years), BMI (kg/m^2^), HbA1c (mmol/mol), esophageal and gastric dysmotility, secondary complications, and symptoms, examined as univariate analysis. No presence of antibodies, symptoms or pathological findings was used as reference. P < 0.05 was considered statistically significant.

## Results

### Patient characteristics

Thirty-nine patients with diabetes mellitus (27 females), age 52.4 ± 12.5 years, were included. All the patients were insulin-treated, and were in acceptable metabolic control (Table 1). Esophageal dysmotility was more common (69%) than gastroparesis (18%). Retinopathy was the most common diabetes complication (72%) (Table 1). Although the patients were included regardless of the presence of gastrointestinal symptoms, the majority of patients reported some sort of symptoms related to the gastrointestinal tract and food intake (Table 2).

**Table 1:** The associations between characteristics in patients with diabetes mellitus and the presence of antibodies against gonadotropin-releasing hormone (GnRH) in serum

	**Patients**	**GnRH IgM −/+**	**GnRH IgG −/+**
	**(N = 39)**	**OR, 95% CI**	**OR, 95% CI**
**Age** (years)	52.4 ± 12.5	1.469 (0.927-1.034)	0.019 (0.964–1.127)
**Gender** (Female/male) n (%)	27(69)/12(31)	1.696 (0.413-1.976)	2.667 (0.452–15.722)
**Type of diabetes** (1/2)	36/3	1.00 (0.082–12.164)	3.100 (0.235–40.895)
**Duration of the disease** (years)	26.3 ± 13.1	0.654 (0.940–1.042)	0.048 (0.974–1.126)
**BMI** (kg/m^2^)	26.5 ± 5.8	0.835 (0.699–0.998)	1.103 (0.958–1.269)
**HbA1c** (mmol/mol)	65.9 ± 9.9	0.983 (0.917–1.053)	0.968 (0.880–1.063)
**Esophageal dysmotility** n (%)			
No (reference)	12 (31)	1.000	1.000
Yes	27 (69)	0.589 (0.143–2.424)	2.500 (0.259–24.096)
**Gastric dysmotility** n (%)			
No (reference)	32 (82)	1.000	-
Yes	7 (18)	3.407 (0.633–18.35)	-
**Retinopathy** n (%)			
No (reference)	11 (28)	1.000	1.000
Yes	28 (72)	0.829 (0.192–3.577)	2.174 (0.224–21.079)
**Angiopathy** n (%)			
No (reference)	26 (67)	1.000	1.000
Yes	13 (33)	0.480 (0.106–2.179)	0.350 (0.036–3.358)
**Microalbuminuria** n (%)			
No (reference)	35 (90)	-	1.000
Yes	4 (10)	-	7.750 (0.842–71.314)
**Macroalbuminuria** n (%)			
No (reference)	36 (92)	1.000	1.000
Yes	3 (8)	4.545 (0.372–55.542)	3.100 (0.235–40.895)
**Autonomic neuropathy** n (%)			
No (reference)	29 (74)	1.000	1.000
Yes	10 (26)	1.481 (0.334–6.572)	9.000 (1.327–61.025)
**Peripheral neuropathy** n (%)			
No (reference)	20 (51)	1.000	1.000
Yes	19 (49)	0.857 (0.226–3.254)	1.062 (0.187–6.052)

*BMI* Body mass index, *HbA1c* Glycosylated hemoglobin. Values are given as mean ± standard deviation (SD) or n (%). *OR* Odds ratio, *CI* Confidence interval, - = calculations not possible to carry out due to a low number of patients. In the statistical calculations, patients were divided into those with and without (reference) antibodies.

**Table 2:** The association between various symptoms in patients with diabetes mellitus and the presence of antibodies against gonadotropin-releasing hormone (GnRH) in serum

**Symptoms**	**Patients (N = 39)**	**GnRH IgM −/+**	**GnRH IgG −/+**
	**n (%)**	**OR, 95% CI**	**OR, 95% CI**
**Loss of appetite**			
No (reference)	31 (80)	1.000	1.000
Yes	8 (20)	4.792 (0.928–24.750)	0.743 (0.074–7.436)
**Swallowing disturbances**			
No (reference)	32 (82)	1.000	1.000
Yes	7 (18)	0.764 (0.127–4.596)	0.900 (0.088–9.178)
**Meal-related cough**			
No (reference)	33 (85)	1.000	1.000
Yes	6 (15)	1.000 (0.158–6.330)	3.625 (0.494–26.611)
**Early satiety**			
No (reference)	24 (62)	1.000	1.000
Yes	15 (38)	0.606 (0.148–2.486)	0.769 (0.123–4.821)
**Nausea**			
No (reference)	28 (72)	1.000	1.000
Yes	11 (28)	0.343 (0.062–1.898)	0.460 (0.047–4.460)
**Abdominal fullness**			
No (reference)	23 (59)	1.000	1.000
Yes	16 (41)	0.300 (0.067–1.347)	0.240 (0.025–2.286)
**Bloating**			
No (reference)	19 (49)	1.000	1.000
Yes	20 (51)	0.278 (0.067–1.147)	0.941 (0.165–5.361)
**Regurgitation**			
No (reference)	27 (69)	1.000	1.000
Yes	12 (31)	0.114 (0.013–1.009)	2.667 (0.452–15.722)
**Constipation**			
No (reference)	28 (72)	1.000	1.000
Yes	11 (28)	0.675 (0.145–3.135)	0.460 (0.047–4.460)
**Diarrhea**			
No (reference)	33 (85)	1.000	1.000
Yes	6 (15)	1.000 (0.158–6.330)	1.120 (0.107–11.726)
**Symptomatic postprandial hypoglycemia**			
No (reference)	30 (77)	1.000	1.000
Yes	9 (23)	0.764 (0.127–4.596)	0.900 (0.088–9.178)
**Postprandial perspiration**			
No (reference)	34 (87)	1.000	1.000
Yes	5 (13)	1.394 (0.203–9.585)	1.450 (0.133–15.793)

*n* number, *OR* Odds ratio, *CI* Confidence interval. In the statistical calculations, patients were divided into those with and without (reference) antibodies.

### Antibodies against gonadotropin-releasing hormone

The prevalence of IgM antibodies in patients with diabetes mellitus was 33%, to be compared with 14% in controls (p = 0.027), with a higher titer level in the patients (p = 0.000). The prevalence of IgG antibodies in patients was 15% and none in the controls (p = 0.000) (Table 3). There was no association between the expression of IgM and IgG antibodies (p = 0.631), but one or both of these antibodies were found in 17 of 39 patients (44%) compared with 11 of 78 controls (14%) (p = 0.001). None of the secondary complications or symptoms was associated with the presence of IgM antibodies (Tables 1 and 2). The prevalence of vomiting (n = 2), weight loss (n = 1), and evacuation incontinence (n = 2) was too low to allow calculations. The only variable associated with IgM antibodies was BMI, with lower BMI being associated with an increased prevalence of antibodies (OR = 0.835, 95% CI = 0.699–0.998). Presence of autonomic neuropathy was the only variable associated with IgG antibodies (OR = 9.000, 95% CI = 1.327–61.025). Esophageal dysmotility or gastroparesis were not associated with the presence of IgM antibodies (OR = 0.589, 95% CI = 0.143–2.424 and OR = 3.407, 95% CI = 0.633–18.350, respectively). Neither was esophageal dysmotility associated with the presence of IgG antibodies (OR = 2.500, 95% CI = 0.259–24.096). Association between gastroparesis and the presence of IgG antibodies was not able to calculate (Table 1). There were no differences in gastric emptying rate or age between patients who did or did not express IgM or IgG antibodies (p = 0.059 vs. p = 0.538 and p = 0.466 vs. p = 0.303, respectively). There was no correlation between the level of IgM or IgG antibodies and gastric emptying rate (p = 0.602 and p = 0.700, respectively). Neither did the level of IgM or IgG antibodies correlate to esophageal aperistalsis (p = 0.268 and p = 0.518, respectively), mean peristaltic contraction amplitude (p = 0.275 and p = 0.156, respectively), percentage of simultaneous, non-propulsive peristaltic waves (p = 0.955 and p = 0.441, respectively), speed of the peristaltic wave (p = 0.603 and p = 0.493, respectively) or resting pressure in the lower esophageal sphincter (p = 0.562 and p = 0.827, respectively).

**Table 3:** Levels of antibodies against gonadotropin-releasing hormone (GnRH) in serum in patients and controls

	**Diabetes mellitus**	**Controls**	**P-value**
	**N = 39**	**n = 78**	
**Prevalence IgG antibodies n** (%)	6 (15)	0	0.000
**Level of IgG antibodies** (RU)	2.4 (0.4-1.9)	*	-
**Prevalence IgM antibodies n** (%)	13 (33)	11 (14)	0.027
**Level of IgM antibodies** (RU)	1.2 (0.6-5.0)	0.2 (0.1-0.3)	0.000

Antibody levels are presented as relative units (RU), median (interquartile range). n (%) = number and percentage of subjects. * = no IgG antibodies were found. Mann-Whitney *U-* test or Fisher’s exact test. P < 0.05 was considered statistically significant.

## Discussion

The present study showed that patients with diabetes mellitus expressed antibodies against GnRH in serum. The result confirms previous findings of presence of GnRH antibodies in patients expressing a high prevalence of gastrointestinal symptoms and dysmotility and/or diabetes mellitus [17]. Apart from a weak association between the presence of IgM antibodies and lower BMI, and an association between the presence of IgG antibodies and autonomic neuropathy, no other associations with the antibody expression were found.

Gonadotropin-releasing hormone is secreted by the hypothalamus and its most important effect is on the pituitary, stimulating gonadotropin synthesis and secretion. It is a crucial hormone in reproductive physiology and sexual behavior [6]. Peripherally, GnRH and GnRH receptors have been found in the rat myenteric plexus and the intestinal epithelium [22,23], and GnRH has been described in human myenteric neurons [8]. Gonadotropin-releasing hormone has been shown to inhibit the release of gastric secretion and gastrin release in dog [9], and to stimulate motor function in the gastrointestinal tract in female rats and in a patient suffering from chronic intestinal pseudo-obstruction [10,11]. Furthermore, GnRH antibodies have been shown to have anti-proliferative effects on epithelial and smooth muscle cells [12,13] and gastrointestinal tumor cell lines [24,25]. In another study, the GnRH receptor has been found on murine endometrium mediating apoptosis [26]. Thus, GnRH may affect growth regulation of gastrointestinal structures.

We have now repeatedly described that patients with IBS and dysmotility express GnRH antibodies [8,16,17], but the effect of GnRH and/or its antibodies on the physiology and pathophysiology of the gastrointestinal tract remains to be determined. In our previous report, the titer of antibodies, but not the prevalence, was higher in patients compared with controls [17]. This is in contrast with the present findings of an increased prevalence of antibodies in relation to diabetes mellitus. This discrepancy may be explained by the low number of patients examined in the former study, with a greater percentage of females (20 patients, 11 females) [17]. Nevertheless, there was an enhanced expression of GnRH antibodies in both studies in relation to diabetes mellitus. A recent report showed a significant effect of the intestinal GnRH mRNA level on blood glucose levels, indicating that GnRH may be of importance for glucose homeostasis [27]. Moreover, development of diabetes mellitus and autoimmune thyroiditis has been reported after long-term administration of GnRH [28].

In the present study, lower BMI in diabetes mellitus was associated with the expression of IgM antibodies. We have little knowledge about the exact mechanisms of appetite and body weight regulation, but a range of different peptide hormones and neuropeptides seems to be involved [29]. It is recognized that appropriate regulation of reproduction, energy intake, and energy expenditure, and thus maintenance of body weight and fertility, relies on complex hypothalamic neurocircuitry [30]. Food intake and the reproductive function are closely linked. The presence of autoantibodies against melanocyte-stimulating hormone has been documented in healthy man and rat. These antibodies, and other autoantibodies, could be involved in the regulation of feeding, but could also influence the level of anxiety. The authors conclude that autoantibodies against appetite-regulating neuropeptides and peptide hormones may be important participants in mechanisms controlling appetite and food intake [31]. Antibodies against GnRH may pass the blood brain barrier (BBB) and could hypothetically affect appetite-regulating structures in the hypothalamus. Furthermore, the peripheral release of appetite-regulating neuropeptides and peptide hormones from the gastrointestinal tract may be regulated by GnRH present in the ENS [8,22,23,32].

Repeated treatment with GnRH has been shown to render enteric dysmotility and antibodies against GnRH in serum in some cases [14]. The antibodies are assumed to be secondary to the dysmotility, reflecting neuronal damage [14]. Gastrointestinal dysmotility is associated with autonomic neuropathy [33]. Thus, the association between IgG antibodies and autonomic neuropathy in this study may reflect an association between gastrointestinal neuron damage in ENS and autonomic neuropathy. On the contrary, a few studies have reported the presence of antibodies in serum in patients with autonomic neuropathy, so the antibodies may reflect neuronal damage in the autonomic nervous system as well [34,35]. We do not know the difference between the expression of IgM and IgG antibodies in the patients with diabetes mellitus. Probably, the expression of IgM antibodies is persisting in this entity, as the patients had had duration of diabetes mellitus for several years, before inclusion in the study.

One of the limitations of this study is the small sample size. However, we need to perform small pilot studies before testing our hypothesis in greater cohorts with hundreds of patients. Another limitation is that the motility of the lower gastrointestinal tract was not examined, and that the manometry was carried out only over a short time span. If an ambulatory manometry for 24 h had been used, associations may have been found. We chose to examine the patients with as few examinations as possible, to diminish the burden on the patients, and to be able to include more patients. In future studies, it seems more relevant to focus on esophageal manometry examination than on gastric emptying scintigraphy, which seems to be a smaller problem among these patients. The test method needs to be further evaluated concerning the optimal cut-off level. In our ELISA, we considered all relative antibody units above zero as expression of antibodies. If a higher baseline level of antibodies present in serum had been regarded as expression, almost all controls would have been below this cut-off. When more patients have been studied, the cut-off level for positive expression should be redefined.

## Conclusions

Patients with diabetes mellitus express antibodies against GnRH in serum. Apart from weak associations between the presence of IgM antibodies and lower BMI, and IgG antibodies and autonomic neuropathy, no other associations with the antibody expression are found.

## Availability of supporting data

The data supporting the results of this article is included within the article.

## Abbreviations

BMI: Body mass index; ENS: Enteric nervous system; GnRH: Gonadotropin-releasing hormone; HbA1c: Glycosylated hemoglobin; IBS: Irritable bowel syndrome; IQR: Interquartile range; ROI: Region of interest; RT: Room temperature; RU: Relative units.

## Competing interests

The authors declare that they have no competing interests.

## Authors’ contributions

All authors participated in the design of the study. KB and AF collected the blood samples and data from the Department of Endocrinology. KS collected samples from healthy blood donors. RA and GNF performed the ELISA analyses. BO contributed to the statistical analyses and wrote the manuscript. BO supported the study financially (Crafoord and Bengt Ihre Foundations, Konung Gustaf V:s och Drottning Victorias Frimurarestiftelse and Development Foundations of Region Skåne). All authors contributed to the manuscript with constructive criticism, and read and approved the final manuscript.

## References

[B1] Parkman HP Camilleri M Farrugia G McCallum RW Bharucha AE Mayer EA Tack JF Spiller R Horowitz M Vinik AI Galligan JJ Pasricha PJ Kuo B Szarka LA Marciani L Jones K Parrish CR Sandroni P Abell T Ordog T Hasler W Koch KL Sanders K Norton NJ Hamilton F Gastroparesis and functional dyspepsia: excerpts from the AGA/ANMS meeting Neurogastroenterol Motil 2010 22 113 133 10.1111/j.1365-2982.2009.01434.x 20003077 PMC2892213

[B2] Ohlsson B Melander O Thorson O Olsson R Ekberg O Sundkvist G Oesophageal dysmotility, delayed gastric emptying and autonomic neuropathy correlate to disturbed glucose homeostasis Diabetologia 2006 49 2010 2014 10.1007/s00125-006-0354-9 16832660

[B3] Faraj J Melander O Sundkvist G Olsson R Thorsson O Ekberg O Ohlsson B Oesophageal dysmotility, delayed gastric emptying and gastrointestinal symptoms in patients with diabetes mellitus Diabet Med 2007 24 1235 1239 10.1111/j.1464-5491.2007.02236.x 17725632

[B4] Gustafsson RJ Littorin B Berntorp K Frid A Thorsson O Olsson R Ekberg O Ohlsson B Oesophageal dysmotility is more common than gastroparesis in diabetes mellitus Rev Diabet Stud 2011 8 258 265 10.1900/RDS.2011.8.268 PMC3280012 22189550

[B5] Borg J Melander O Johansson L Uvnas-Moberg K Rehfeld JF Ohlsson B Gastroparesis is associated with oxytocin deficiency, oesophageal dysmotility with hyperCCKemia, and autonomic neuropathy with hypergastrinemia BMC Gastroenterol 2009 9 17 10.1186/1471-230X-9-17 19243587 PMC2650701

[B6] Maeda K Ohkura S Uenoyama Y Wakabayashi Y Oka Y Tsukamura H Okamura H Neurobiological mechanisms underlying GnRH pulse generation by the hypothalamus Brain Res 2010 1364 103 115 20951683 10.1016/j.brainres.2010.10.026

[B7] Hazum E Conn PM Molecular mechanism of gonadotropin releasing hormone (GnRH) action, I. The GnRH receptor Endocr Rev 1988 9 379 386 10.1210/edrv-9-4-379 2851440

[B8] Ohlsson B Veress B Ekblad E Montgomery A Janciauskiene S Antibodies against gonadotropin-releasing hormone (GnRH) and destruction of enteric neurons in 3 patients suffering from gastrointestinal dysfunction BMC Gastroenterol 2010 10 48 10.1186/1471-230X-10-48 20487533 PMC2885307

[B9] Soldani G Del Tacca MM Bambini G Polloni A Bernardini C Martinotti E Martino E Effects of gonadotropin-releasing hormone (GnRH) on gastric secretion and gastrin release in the dog J Endocrinol Invest 1982 5 393 396 6132944 10.1007/BF03350539

[B10] Khanna R Browne RM Heiner AD Clench MH Mathias JR Leuprolide acetate affects intestinal motility in female rats before and after ovariectomy Am J Physiol 1992 262 G185 G190 1733265 10.1152/ajpgi.1992.262.1.G185

[B11] Mathias JR Baskin GS Reeves-Darby VG Clench MH Smith LL Calhoon JH Chronic intestinal pseudoobstruction in a patient with heart-lung transplant. Therapeutic effect of leuprolide acetate Dig Dis Sci 1992 37 1761 1768 10.1007/BF01299872 1330462

[B12] Gama P Alvares EP LHRH and somatostatin effects on the cell proliferation of the gastric epithelium of suckling and weaning rats Regul Pept 1996 63 73 78 10.1016/0167-0115(96)00013-4 8837213

[B13] Chen L He HX Sun XD Zhao J Liu LH Huang WQ Zhang R-Q Expression of gonadotropin-releasing hormone receptor and effect of gonadotropin-releasing hormone analogue on proliferation of cultured gastric smooth muscle cells of rats World J Gastroenterol 2004 10 1780 1784 15188505 10.3748/wjg.v10.i12.1780 PMC4572268

[B14] Hammar O Ohlsson B Veress B Alm R Fredrikson GN Montgomery A Depletion of enteric gonadotropin-releasing hormone is found in a few patients suffering from severe gastrointestinal dysmotility Scand J Gastroenterol 2012 47 1165 1173 22835010 10.3109/00365521.2012.706826

[B15] Sand E Voss U Hammar O Alm R Nordin Fredrikson G Ohlsson B Ekblad E Gonadotropin-releasing hormone analog buserelin causes neuronal loss in rat gastrointestinal tract Cell Tissue Res 2013 351 521 534 10.1007/s00441-012-1534-1 23254679

[B16] Ohlsson B Scheja A Janciauskiene S Mandl T Functional bowel symptoms and GnRH antibodies: common findings in patients with primary Sjögren’s syndrome but not in systemic sclerosis Scand J Rheumatol 2009 23 1 2 10.1080/03009740802709069 19308876

[B17] Ohlsson B Sjöberg K Alm R Nordin Fredrikson G Patients with irritable bowel syndrome and dysmotility express antibodies against gonadotropin-releasing hormone in serum Neurogastroenterol Motil 2011 23 1000 1006 10.1111/j.1365-2982.2011.01744.x 21714833

[B18] Hoelzel W Weykamp C Jeppsson JO Miedema K Barr JR Goodall I Hoshino T John WG Kobold U Little R Mosca A Mauri P Paroni R Susanto F Takei I Thienpont L Umemoto M Wiedmeyer HM IFCC Working Group on HbA1c Standardization IFCC reference system for measurement of hemoglobin A1c in human blood and the national standardization schemes in the united states, japan, and sweden: a method-comparison study Clin Chem 2004 50 166 174 10.1373/clinchem.2003.024802 14709644

[B19] Spechler SJ Castell DO Classification of oesophageal motility abnormalities Gut 2001 49 145 151 10.1136/gut.49.1.145 11413123 PMC1728354

[B20] Castell JA Dalton CB Castell DO Online computer analysis of human lower esophageal sphincter relaxation Am J Physiol 1988 255 G794 G799 3202172 10.1152/ajpgi.1988.255.6.G794

[B21] Hanson M Lilja B Gastric emptying in smokers Scand J Gastroenterol 1987 22 1102 1104 10.3109/00365528708991965 3423735

[B22] Ho JS Nagle GT Mathias JR Clench MH Fan X Kalmaz GD Sallustio JE Eaker EY Presence of gonadotropin-releasing hormone (GnRH) receptor mRNA in rat myenteric plexus cells Comp Biochem Physiol B Biochem Mol Biol 1996 113 817 821 10.1016/0305-0491(95)02114-0 8925450

[B23] Huang W Yao B Sun L Pu R Wang L Zhang R Immunohistochemical and in situ hybridization studies of gonadotropin releasing hormone (GnRH) and its receptor in rat digestive tract Life Sci 2001 68 1727 1734 10.1016/S0024-3205(01)00968-7 11270619

[B24] Darro F Camby I Kruczynski A Pasteels JL Martinez J Kiss R Characterisation of the influence of anti-gastrin, anti-epidermal growth factor, anti-oestradiol, and anti-luteinising hormone releasing hormone antibodies on the proliferation of 27 cell lines from the gastrointestinal tract Gut 1995 36 220 230 10.1136/gut.36.2.220 7883221 PMC1382408

[B25] Aguilar-Rojas A Huerta-Reyes M Human gonadotropin-releasing hormone receptor-activated cellular functions and signaling pathways in extra-pituitary tissues and cancer cells (Review) Oncol Rep 2009 22 981 990 19787210 10.3892/or_00000525

[B26] Murdoch WJ Immunolocalization of a gonadotropin-releasing hormone receptor site in murine endometrium that mediates apoptosis Cell Tissue Res 1995 282 527 529 10.1007/BF00318886 8581948

[B27] Wang L Wu J Cao H Chen R Zhang N Fu J Gao B Zhang J Hou R Tang C Ji Q The correlation between intestinal gonadotropin-releasing hormone (GnRH) and proglucagon in hyperlipidemic rats and Goto-Kakizaki (GK) rats Endocrin Pathol 2009 20 227 234 10.1007/s12022-009-9086-y 19669946

[B28] Krstevska-Konstantinova M Jancevska A Gucev Z Autoimmune thyroiditis and diabetes mellitus type 1 after long-term gonadotropin-releasing hormone agonist treatment for central precocious puberty: evolution or coincidence? J Pediatr Endocrinol Metab 2010 23 403 406 20583547 10.1515/jpem.2010.063

[B29] Schloegl H Percik R Horstmann A Villringer A Stumvoll M Peptide hormones regulating appetite–focus on neuroimaging studies in humans Diabetes Metab Res Rev 2011 27 104 112 10.1002/dmrr.1154 21294236

[B30] Small CJ Stanley SA Bloom SR Appetite control and reproduction: leptin and beyond Semin Reprod Med 2002 20 389 398 10.1055/s-2002-36712 12536362

[B31] Fetissov SO Sinno MH Coquerel Q Do Rego JC Coëffier M Gilbert D Gilbert T Déchelotte P Emerging role of autoantibodies against appetite-regulating neuropeptides in eating disorders Nutrition 2008 24 854 859 10.1016/j.nut.2008.06.021 18725083

[B32] Ukkola O Peripheral regulation of food intake: new insights J Endocrinol Invest 2004 27 96 98 15053251 10.1007/BF03350918

[B33] Mattsson T Roos R Sundkvist G Valind S Ohlsson B Sympathetic nerve dysfunction is common in patients with chronic intestinal pseudo-obstruction J Clin Gastroenterol 2008 42 174 177 10.1097/01.mcg.0000225649.54566.02 18209588

[B34] Ejskjaer N Arif S Dodds W Zanone MM Vergani D Watkins PJ Peakman M Prevalence of autoantibodies to autonomic nervous tissue structures in type 1 diabetes mellitus Diabet Med 1999 16 544 549 10.1046/j.1464-5491.1999.00092.x 10445828

[B35] Granberg V Ejskjaer N Peakman M Sundkvist G Autoantibodies to autonomic nerves associated with cardiac and peripheral autonomic neuropathy Diabetes Care 2005 28 1959 1964 10.2337/diacare.28.8.1959 16043739

